# Developmental Fluoride Neurotoxicity: A Systematic Review and Meta-Analysis

**DOI:** 10.1289/ehp.1104912

**Published:** 2012-07-20

**Authors:** Anna L. Choi, Guifan Sun, Ying Zhang, Philippe Grandjean

**Affiliations:** 1 Department of Environmental Health, Harvard School of Public Health, Boston, Massachusetts, USA; 2 School of Public Health, China Medical University, Shenyang, China; 3 School of Stomatology, China Medical University, Shenyang, China; 4 Institute of Public Health, University of Southern Denmark, Odense, Denmark

**Keywords:** fluoride, intelligence, neurotoxicity

## Abstract

Background: Although fluoride may cause neurotoxicity in animal models and acute fluoride poisoning causes neurotoxicity in adults, very little is known of its effects on children’s neurodevelopment.

Objective: We performed a systematic review and meta-analysis of published studies to investigate the effects of increased fluoride exposure and delayed neurobehavioral development.

Methods: We searched the MEDLINE, EMBASE, Water Resources Abstracts, and TOXNET databases through 2011 for eligible studies. We also searched the China National Knowledge Infrastructure (CNKI) database, because many studies on fluoride neurotoxicity have been published in Chinese journals only. In total, we identified 27 eligible epidemiological studies with high and reference exposures, end points of IQ scores, or related cognitive function measures with means and variances for the two exposure groups. Using random-effects models, we estimated the standardized mean difference between exposed and reference groups across all studies. We conducted sensitivity analyses restricted to studies using the same outcome assessment and having drinking-water fluoride as the only exposure. We performed the Cochran test for heterogeneity between studies, Begg’s funnel plot, and Egger test to assess publication bias, and conducted meta-regressions to explore sources of variation in mean differences among the studies.

Results: The standardized weighted mean difference in IQ score between exposed and reference populations was –0.45 (95% confidence interval: –0.56, –0.35) using a random-effects model. Thus, children in high-fluoride areas had significantly lower IQ scores than those who lived in low-fluoride areas. Subgroup and sensitivity analyses also indicated inverse associations, although the substantial heterogeneity did not appear to decrease.

Conclusions: The results support the possibility of an adverse effect of high fluoride exposure on children’s neurodevelopment. Future research should include detailed individual-level information on prenatal exposure, neurobehavioral performance, and covariates for adjustment.

A recent report from the National Research Council (NRC 2006) concluded that adverse effects of high fluoride concentrations in drinking water may be of concern and that additional research is warranted. Fluoride may cause neurotoxicity in laboratory animals, including effects on learning and memory (Chioca et al. 2008; Mullenix et al. 1995). A recent experimental study where the rat hippocampal neurons were incubated with various concentrations (20 mg/L, 40 mg/L, and 80 mg/L) of sodium fluoride *in vitro* showed that fluoride neurotoxicity may target hippocampal neurons (Zhang M et al. 2008). Although acute fluoride poisoning may be neurotoxic to adults, most of the epidemiological information available on associations with children’s neurodevelopment is from China, where fluoride generally occurs in drinking water as a natural contaminant, and the concentration depends on local geological conditions. In many rural communities in China, populations with high exposure to fluoride in local drinking-water sources may reside in close proximity to populations without high exposure (NRC 2006).

Opportunities for epidemiological studies depend on the existence of comparable population groups exposed to different levels of fluoride from drinking water. Such circumstances are difficult to find in many industrialized countries, because fluoride concentrations in community water are usually no higher than 1 mg/L, even when fluoride is added to water supplies as a public health measure to reduce tooth decay. Multiple epidemiological studies of developmental fluoride neurotoxicity were conducted in China because of the high fluoride concentrations that are substantially above 1 mg/L in well water in many rural communities, although microbiologically safe water has been accessible to many rural households as a result of the recent 5-year plan (2001–2005) by the Chinese government. It is projected that all rural residents will have access to safe public drinking water by 2020 (World Bank 2006). However, results of the published studies have not been widely disseminated. Four studies published in English (Li XS et al. 1995; Lu et al. 2000; Xiang et al. 2003; Zhao et al. 1996) were cited in a recent report from the NRC (2006), whereas the World Health Organization (2002) has considered only two (Li XS et al. 1995; Zhao et al. 1996) in its most recent monograph on fluoride.

Fluoride readily crosses the placenta (Agency for Toxic Substances and Disease Registry 2003). Fluoride exposure to the developing brain, which is much more susceptible to injury caused by toxicants than is the mature brain, may possibly lead to permanent damage (Grandjean and Landrigan 2006). In response to the recommendation of the NRC (2006), the U.S. Department of Health and Human Services (DHHS) and the U.S. EPA recently announced that DHHS is proposing to change the recommended level of fluoride in drinking water to 0.7 mg/L from the currently recommended range of 0.7–1.2 mg/L, and the U.S. EPA is reviewing the maximum amount of fluoride allowed in drinking water, which currently is set at 4.0 mg/L (U.S. EPA 2011).

To summarize the available literature, we performed a systematic review and meta-analysis of published studies on increased fluoride exposure in drinking water associated with neurodevelopmental delays. We specifically targeted studies carried out in rural China that have not been widely disseminated, thus complementing the studies that have been included in previous reviews and risk assessment reports.

## Methods

*Search strategy.* We searched MEDLINE (National Library of Medicine, Bethesda, MD, USA; http://www.ncbi.nlm.nih.gov/pubmed), Embase (Elsevier B.V., Amsterdam, the Netherlands; http://www.embase.com), Water Resources Abstracts (Proquest, Ann Arbor, MI, USA; http://www.csa.com/factsheets/water-resources-set-c.php), and TOXNET (Toxicology Data Network; National Library of Medicine, Bethesda, MD, USA; http://toxnet.nlm.nih.gov) databases to identify studies of drinking-water fluoride and neurodevelopmental outcomes in children. In addition, we searched the China National Knowledge Infrastructure (CNKI; Beijing, China; http://www.cnki.net) database to identify studies published in Chinese journals only. Key words included combinations of “fluoride” or “drinking water fluoride,” “children,” “neurodevelopment” or “neurologic” or “intelligence” or “IQ.” We also used references cited in the articles identified. We searched records for 1980–2011. Our literature search identified 39 studies, among which 36 (92.3%) were studies with high and reference exposure groups, and 3 (7.7%) studies were based on individual-level measure of exposures. The latter showed that dose-related deficits were found, but the studies were excluded because our meta-analysis focused on studies with the high- and low-exposure groups only. In addition, two studies were published twice, and the duplicates were excluded.

*Inclusion criteria and data extraction.* The criteria for inclusion of studies included studies with high and reference fluoride exposures, end points of IQ scores or other related cognitive function measures, presentation of a mean outcome measure, and associated measure of variance [95% confidence intervals (CIs) or SEs and numbers of participants]. Interpretations of statistical significance are based on an alpha level of 0.05. Information included for each study also included the first author, location of the study, year of publication, and numbers of participants in high-fluoride and low-fluoride areas. We noted and recorded the information on age and sex of children, and parental education and income if available.

*Statistical analysis.* We used STATA (version 11.0; StataCorp, College Station, TX, USA) and available commands (Stern 2009) for the meta-analyses. A standardized weighted mean difference (SMD) was computed using both fixed-effects and random-effects models. The fixed-effects model uses the Mantel–Haenszel method assuming homogeneity among the studies, whereas the random-effects model uses the DerSimonian and Laird method, incorporating both a within-study and an additive between-studies component of variance when there is between-study heterogeneity (Egger et al. 2001). The estimate of the between-study variation is incorporated into both the SE of the estimate of the common effect and the weight of individual studies, which was calculated as the inverse sum of the within and between study variance. We evaluated heterogeneity among studies using the *I*^2^ statistic, which represents the percentage of total variation across all studies due to between-study heterogeneity (Higgins and Thompson 2002). We evaluated the potential for publication bias using Begg and Egger tests and visual inspection of a Begg funnel plot (Begg and Mazumdar 1994; Egger et al. 1997). We also conducted independent meta-regressions to estimate the contribution of study characteristics (mean age in years from the age range and year of publication in each study) to heterogeneity among the studies. The scoring standard for the Combined Raven’s Test–The Rural edition in China (CRT-RC) test classifies scores of ≤ 69 and 70–79 as low and marginal intelligence, respectively (Wang D et al. 1989). We also used the random-effects models to estimate risk ratios for the association between fluoride exposure and a low/marginal versus normal Raven’s test score among children in studies that used the CRT-RC test (Wang D et al. 1989). Scores indicating low and marginal intelligence (≤ 69 and 70–79, respectively) were combined as a single outcome due to small numbers of children in each outcome subgroup.

## Results

**Figure 1:**
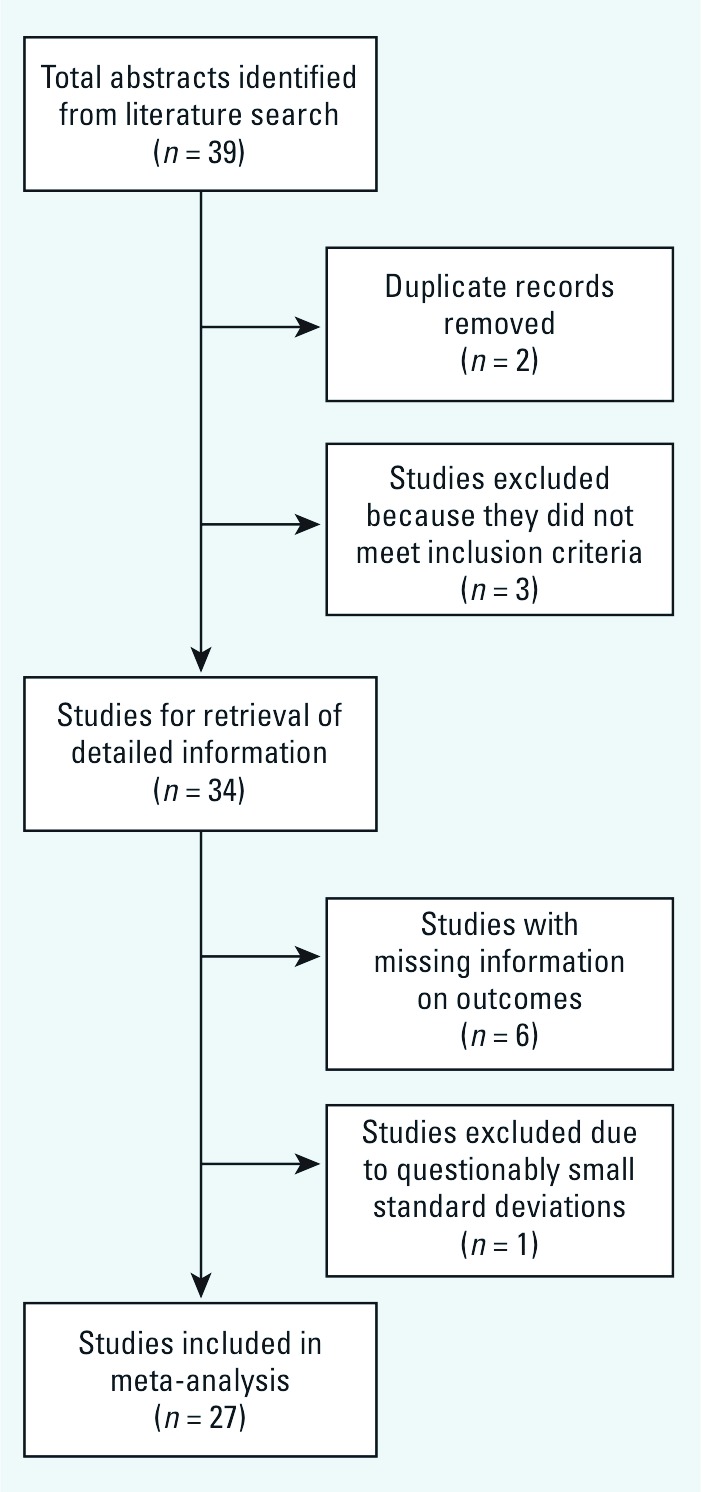
Flow diagram of the meta-analysis.

Six of the 34 studies identified were excluded because of missing information on the number of subjects or the mean and variance of the outcome [see Figure 1 for a study selection flow chart and Supplemental Material, Table S1 (http://dx.doi.org/10.1289/ehp.1104912) for additional information on studies that were excluded from the analysis]. Another study (Trivedi et al. 2007) was excluded because SDs reported for the outcome parameter were questionably small (1.13 for the high-fluoride group, and 1.23 for the low-fluoride group) and the SMD (–10.8; 95% CI: –11.9, –9.6) was > 10 times lower than the second smallest SMD (–0.95; 95% CI: –1.16, –0.75) and 150 times lower than the largest SMD (0.07; 95% CI: –0.083, 0.22) reported for the other studies, which had relatively consistent SMD estimates. Inclusion of this study in the meta-analysis resulted with a much smaller pooled random-effects SMD estimate and a much larger *I*^2^ (–0.63; 95% CI: –0.83, –0.44, *I*^2^ 94.1%) compared with the estimates that excluded this study (–0.45; 95% CI: –0.56, –0.34, *I*^2^ 80%) (see Supplemental Material, Figure S1). Characteristics of the 27 studies included are shown in Table 1 (An et al. 1992; Chen et al. 1991; Fan et al. 2007; Guo et al. 1991; Hong et al. 2001; Li FH et al. 2009; Li XH et al. 2010; Li XS 1995; Li Y et al. 1994; Li Y et al. 2003; Lin et al. 1991; Lu et al. 2000; Poureslami et al. 2011; Ren et al. 1989; Seraj et al. 2006; Sun et al. 1991; Wang G et al. 1996; Wang SH et al. 2001; Wang SX et al. 2007; Wang ZH et al. 2006; Xiang et al. 2003; Xu et al. 1994; Yang et al. 1994; Yao et al. 1996, 1997; Zhang JW et al. 1998; Zhao et al. 1996). Two of the studies included in the analysis were conducted in Iran (Poureslami et al. 2011; Seraj et al. 2006); the other study cohorts were populations from China. Two cohorts were exposed to fluoride from coal burning (Guo et al. 1991; Li XH et al. 2010); otherwise populations were exposed to fluoride through drinking water. The CRT-RC was used to measure the children’s intelligence in 16 studies. Other intelligence measures included the Wechsler Intelligence tests (3 studies; An et al. 1992; Ren et al. 1989; Wang ZH et al. 1996), Binet IQ test (2 studies; Guo et al. 1991; Xu et al. 1994), Raven’s test (2 studies; Poureslami et al. 2011; Seraj et al. 2006), Japan IQ test (2 studies; Sun et al. 1991; Zhang JW et al. 1998), Chinese comparative intelligence test (1 study; Yang et al. 1994), and the mental work capacity index (1 study; Li Y et al. 1994). Because each of the intelligence tests used is designed to measure general intelligence, we used data from all eligible studies to estimate the possible effects of fluoride exposure on general intelligence.

**Table 1:** Characteristics of epidemiological studies of fluoride exposure and children’s cognitive outcomes.

Reference	Study location	No. in high- exposure group	No. in reference group	Age range (years)	Fluoride exposure			Outcome measure	Results
Assessment	Range
Ren et al. 1989	Shandong, China	160	169	8–14	High-/low-fluoride villages	Not specified	Wechsler Intelligence testa	Children in high-fluoride region had lower IQ scores
Chen et al. 1991	Shanxi, China	320	320	7–14	Drinking water	4.55 mg/L (high); 0.89 mg/L (reference)	CRT-RCb	The average IQ of children from high-fluoride area were lower than that of the reference area
Guo et al. 1991	Hunan, China	60	61	7–13	Fluoride in coal burning	118.1–1361.7 mg/kg (coal burning area); Control area used wood	Chinese Binetc	Average IQ in fluoride coal-burning area was lower than that in the reference area
Lin et al. 1991	Xinjiang, China	33	86	7–14	Drinking water	0.88 mg/L (high); 0.34 mg/L (reference)	CRT-RCb	Children in the high-fluoride (low-iodine) area had lower IQ scores compared with the children from the reference fluoride (low-iodine) areas
Sun et al. 1991	Guiyang, China	196	224	6.5–12	Rate of fluorosis	Fluorosis: 98.36% (high); not specified (reference)	Japan IQ testd	Mean IQ was lower in all age groups except ≤ 7 years in the area with high fluoride and aluminum (limited to high-fluoride population only)
An et al. 1992	Inner Mongolia, China	121	121	7–16	Drinking water	2.1–7.6 mg/L (high); 0.6–1.0 mg/L (reference)	Wechsler Intelligence testa	IQ scores of children in high-fluoride areas were significantly lower than those of children living in reference fluoride area
Li Y et al. 1994	Sichuan, China	106	49	12–13	Burning of high-fluoride coal to cook grain in high-fluoride area	4.7–31.6 mg/kg (high); 0.5 mg/kg (reference)	Child mental work capacity	Early, prolonged high fluoride intake causes a decrease in the child’s mental work capacity
Xu et al. 1994	Shandong, China	97	32	8–14	Drinking water	1.8 mg/L (high); 0.8 mg/L (reference)	Binet-Simone	Children had lower IQ scores in high-fluoride area than those who lived in the reference area.
Yang et al. 1994	Shandong, China	30	30	8–14	Well water	2.97 mg/L (high); 0.5 mg/L (reference)	Chinese comparative intelligence testf	The average IQ scores was lower in children from high-fluoride and -iodine area than those from the reference area, but the results were not significant
Li XS et al. 1995	Guizhou, China	681	226	8–13	Urine, Dental Fluorosis Index	1.81–2.69 mg/L (high); 1.02 mg/L (reference); DFI 0.8–3.2 (high); DFI < 0.4 (reference)	CRT-RCb	Children living in fluorosis areas had lower IQ scores than children living in nonfluorosis areas
Wang G et al. 1996	Xinjiang, China	147	83	4–7	Drinking water	> 1.0–8.6 mg/L (high); 0.58–1.0 mg/L (reference)	Wechsler Intelligence testa	Average IQ score was lower in children in the high-fluoride group than those in the reference group
Yao et al. 1996	Liaoning, China	266	270	8–12	Drinking water	2–11mg/L (high); 1 mg/L (reference)	CRT-RCb	Average IQ scores of children residing in exposed fluoride areas were lower than those in the reference area
Zhao et al. 1996	Shanxi, China	160	160	7–14	Drinking water	4.12 mg/L (high); 0.91 mg/L (reference)	CRT-RCb	Children living in high-fluoride and -arsenic area had significantly lower IQ scores than those living in the reference fluoride (and no arsenic) area
Yao et al. 1997	Liaoning, China	188	314	7–14	Drinking water	2 mg/L (exposed); 0.4 mg/L (reference)	CRT-RCb	IQ scores of children in the high-fluoride area were lower than those of children in the reference area
Assessment	Range
Zhang JW et al. 1998	Xinjiang, China	51	52	4–10	Drinking water	Not specified	Japan IQ Testd	Average IQ scores of children residing in high-fluoride and -arsenic area were lower than those who resided in the reference area
Lu et al. 2000	Tianjin, China	60	58	10–12	Drinking water	3.15 mg/L (high); 0.37 mg/L (reference)	CRT-RCb	Children in the high-fluoride area scored significantly lower IQ scores than those in the reference area
Hong et al. 2001	Shandong, China	85	32	8–14	Drinking water	2.90 mg/L (high); 0.75 mg/L (reference)	CRT-RCb	Average IQ scores were significantly lower in high-fluoride group (and -iodine) than the reference group
Wang SH et al. 2001	Shandong, China	30	30	8–12	Drinking water	2.97 mg/L (high); 0.5 mg/L (reference)	CRT-RCb	No significant difference in IQ scores of children in the high-fluoride/high-iodine and reference fluoride/low-iodine areas
Li Y et al. 2003	Inner Mongolia, China	720	236	6–13	Fluorosis	Endemic vs. control regions defined by the Chinese Geological Office	CRT-RCb	Average IQ of children in high-fluorosis area was lower than that in the reference area
Xiang et al. 2003	Jiangsu, China	222	290	8–13	Drinking water	0.57–4.5 mg/L (high); 0.18–0.76 mg/L (reference)	CRT-RCb	Mean IQ score was significantly lower in children who lived in the high-fluoride area than that of children in the reference exposure area (both areas also had arsenic exposure)
Seraj et al. 2006	Tehran, Iran	41	85	Not specified	Drinking water	2.5 mg/L (high); 0.4 mg/L (reference)	Raveng	The mean IQ of children in the high-fluoride area was significantly lower than that from the reference fluoride area
Wang ZH et al. 2006	Shanxi, China	202	166	8–12	Drinking water	5.54 ± 3.88 mg/L (high); 0.73 ± 0.28 mg/L (reference)	CRT-RCb	The IQ scores of children in the high-fluoride group were significantly lower than those in the reference group
Fan et al 2007	Shaanxi, China	42	37	7–14	Drinking water	1.14–6.09 mg/L (high); 1.33–2.35 mg/L (reference)	CRT-RCb	The average IQ scores of children residing in the high-fluoride area were lower than those of children residing in the reference area
Wang SX et al. 2007	Shanxi, China	253	196	8–12	Drinking water and urine	3.8–11.5 mg/L (water, high); 1.6–11 mg/L (urine, high); 0.2–1.1 mg/L (water, reference); 0.4–3.9 mg/L (urine, reference)		CRT-RCb	Mean IQ scores were significantly lower in the high-fluoride group than from the reference group in the fluoride/arsenic areas
Li et al. 2009	Hunan, China	60	20	8–12	Coal burning	1.24–2.34 mg/L (high); 0.962 mg/L (reference)	CRT-RCb	Mean IQ was lower in children in coal-burning areas compared to those in the reference group
Li FH et al. 2010	Henan, China	347	329	7–10	Drinking water	2.47 ± 0.75 mg/L (high)	CRT-RCb	No significant difference in IQ scores between children in the exposed and reference groups
Poureslami et al. 2011	Iran	59	60	6–9	Drinking Water	2.38 mg/L (high); 0.41 mg/L (reference)	Raveng	Children in the high-fluoride group scored significantly lower than those in reference group
aWechsler Intelligence Scale (Lin and Zhang 1986). bCRT-RC, Chinese Standardized Raven Test, rural version (Wang G et al. 1989). cChinese Binet Test (Wu 1936). dJapan test (Zhang J et al. 1985). eBinet-Simon Test (Binet and Simon 1922). fChinese comparative intelligence test (Wu 1983). gRaven test (Raven et al. 2003).														

In addition, we conducted a sensitivity analysis restricted to studies that used similar tests to measure the outcome (specifically, the CRT-RC, Wechsler Intelligence test, Binet IQ test, or Raven’s test), and an analysis restricted to studies that used the CRT-RC. We also performed an analysis that excluded studies with co-exposures including iodine and arsenic, or with non-drinking-water fluoride exposure from coal burning.

**Figure 2:**
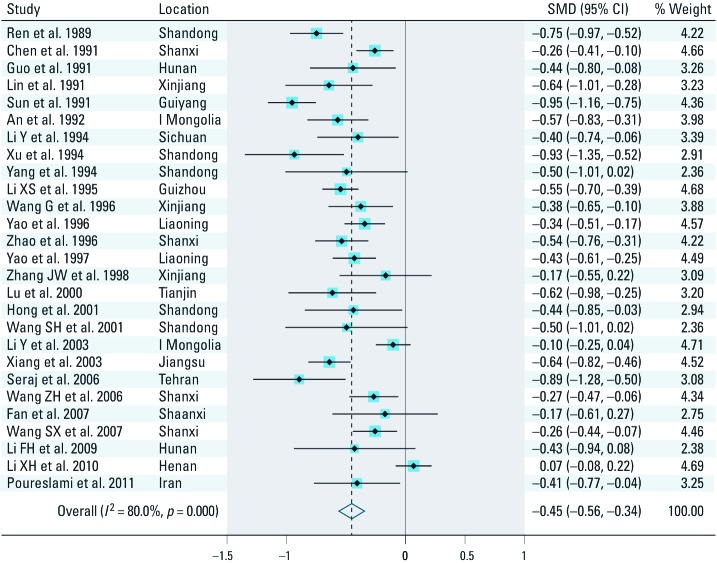
Random-effect standardized weighted mean difference (SMD) estimates and 95% CIs of child’s intelligence score associated with high exposure to fluoride. SMs for individual studies are shown as solid diamonds (♦), and the pooled SMD is shown as an open diamond (◊). Horizontal lines represent 95% CIs for the study-specific SMDs.

*Pooled SMD estimates.* Among the 27 studies, all but one study showed random-effect SMD estimates that indicated an inverse association, ranging from –0.95 (95% CI: –1.16, –0.75) to –0.10 (95% CI: –0.25, 0.04) (Figure 2). The study with a positive association reported an SMD estimate of 0.07 (95% CI: –0.8, 0.22). Similar results were found with the fixed-effects SMD estimates. The fixed-effects pooled SMD estimate was –0.40 (95% CI: –0.44, –0.35), with a *p*-value < 0.001 for the test for homogeneity. The random-effects SMD estimate was –0.45 (95% CI: –0.56, –0.34) with an *I*^2^ of 80% and homogeneity test *p*-value < 0.001 (Figure 2). Because of heterogeneity (excess variability) between study results, we used primarily the random-effects model for subsequent sensitivity analyses, which is generally considered to be the more conservative method (Egger et al. 2001). Among the restricted sets of intelligence tests, the SMD for the model with only CRT-RC tests and drinking-water exposure (and to a lesser extent the model with only CRT-RC tests) was lower than that for all studies combined, although the difference did not appear to be significant. Heterogeneity, however, remained at a similar magnitude when the analyses were restricted (Table 2).

**Table 2:** Sensitivity analyses of pooled random-effects standardized weighted mean difference (SMD) estimates of child’s intelligence score with high exposure of fluoride.

Model	Available studies for analysis	SMD (95% CI)	I^2^	*p*-Value test of heterogeneity
1. Exclude nonstandardized testsa	23	–0.44 (–0.54, –0.33)	77.6%	< 0.001
2. Exclude non–CRT-RC Testsb	16	–0.36 (–0.48, –0.25)	77.8%	< 0.001
3. Exclude studies with other exposures (iodine, arsenic)c or non-drinking-water fluoride exposured	9	–0.29 (–0.44, –0.14)	81.8%	< 0.001
aMental work capacity (Li Y et al. 1994); Japan IQ (Sun et al. 1991; Zhang JW et al. 1998); Chinese comparative scale of intelligence test (Yang et al. 1994). bWechsler intelligence test (An et al. 1992; Ren et al. 1989; Wang G et al. 1996); Chinese Binet IQ (Guo et al. 1991); Raven (Poureslami et al. 2011; Seraj et al. 2006); Binet-Simon (Xu et al. 1994). cIodine (Hong et al. 2001; Lin et al. 1991; Wang SH et al. 2001); arsenic [Wang SX et al. 2007; Xiang et al. 2003; Zhao et al. 1996; (Zhang JW et al. 1998 was already excluded, see note a)]. dFluoride from coal burning [Li FH et al. 2009 (Guo et al. 1991 and Li Y et al. 1994 were already excluded; see notes a and b)].						

*Sources of heterogeneity.* We performed meta-regression models to assess study characteristics as potential predictors of effect. Information on the child’s sex and parental education were not reported in > 80% of the studies, and only 7% of the studies reported household income. These variables were therefore not included in the models. Among the two covariates, year of publication (0.02; 95% CI: 0.006, 0.03), but not mean age of the study children (–0.02; 95% CI: –0.094, 0.04), was a significant predictor in the model with all 27 studies included. *I*^2^ residual 68.7% represented the proportion of residual between-study variation due to heterogeneity. From the adjusted *R*^2^, 39.8% of between-study variance was explained by the two covariates. The overall test of the covariates was significant (*p* = 0.004).

When the model was restricted to the 16 studies that used the CRT-RC, the child’s age (but not year of publication) was a significant predictor of the SMD. The *R*^2^ of 65.6% of between-study variance was explained by the two covariates, and only 47.3% of the residual variation was attributable to heterogeneity. The overall test of both covariates in the model remained significant (*p* = 0.0053). On further restriction of the model to exclude the 7 studies with arsenic and iodine as co-exposures and fluoride originating from coal burning (thus including only the 9 with fluoride exposure from drinking water), neither age nor year of publication was a significant predictor, and the overall test of covariates was less important (*p* = 0.062), in accordance with the similarity of intelligence test outcomes and the source of exposure in the studies included. Although official reports of lead concentrations in the study villages in China were not available, some studies reported high percentage (95–100%) of low lead exposure (less than the standard of 0.01 mg/L) in drinking-water samples in villages from several study provinces (Bi et al. 2010; Peng et al. 2008; Sun 2010).

**Figure 3:**
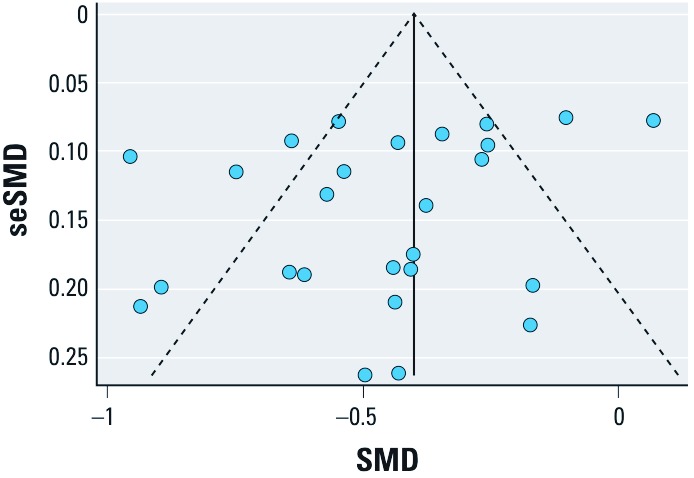
Begg’s funnel plot showing individual studies included in the analysis according to random-effect standardized weighted mean difference (SMD) estimates (*x*-axis) and the SE (se) of each study-specific SMD (*y*-axis). The solid vertical line indicates the pooled SMD estimate for all studies combined and the dashed lines indicated pseudo 95% confidence limits around the pooled SMD estimate.

*Publication bias.* A Begg’s funnel plot with the SE of SMD from each study plotted against its corresponding SMD did not show clear evidence of asymmetry, although two studies with a large SE also reported relatively large effect estimates, which may be consistent with publication bias or heterogeneity (Figure 3). The plot appears symmetrical for studies with larger SE, but with substantial variation in SMD among the more precise studies, consistent with the heterogeneity observed among the studies included in the analysis. Begg (*p* = 0.22) and Egger (*p* = 0.11) tests did not indicate significant (*p* < 0.05) departures from symmetry.

*Pooled risk ratios.* The relative risk (RR) of a low/marginal score on the CRT-RC test (< 80) among children with high fluoride exposure compared with those with low exposure (16 studies total) was 1.93 (95% CI: 1.46, 2.55; *I*^2^ 58.5%). When the model was restricted to 9 studies that used the CRT-RC and included only drinking-water fluoride exposure (Chen et al. 1991; Fan et al. 2007; Li XH et al. 2010; Li XS et al. 1995; Li Y et al. 2003; Lu et al. 2000; Wang ZH et al. 2006; Yao et al. 1996, 1997), the estimate was similar (RR = 1.75; 95% CI: 1.16, 2.65; *I*^2^ 70.6%). Although fluoride exposure showed inverse associations with test scores, the available exposure information did not allow a formal dose–response analysis. However, dose-related differences in test scores occurred at a wide range of water-fluoride concentrations.

## Discussion

Findings from our meta-analyses of 27 studies published over 22 years suggest an inverse association between high fluoride exposure and children’s intelligence. Children who lived in areas with high fluoride exposure had lower IQ scores than those who lived in low-exposure or control areas. Our findings are consistent with an earlier review (Tang et al. 2008), although ours more systematically addressed study selection and exclusion information, and was more comprehensive in *a*) including 9 additional studies, *b*) performing meta-regression to estimate the contribution of study characteristics as sources of heterogeneity, and *c*) estimating pooled risk ratios for the association between fluoride exposure and a low/marginal Raven’s test score.

As noted by the NRC committee (NRC 2006), assessments of fluoride safety have relied on incomplete information on potential risks. In regard to developmental neurotoxicity, much information has in fact been published, although mainly as short reports in Chinese that have not been available to most expert committees. We carried out an extensive review that includes epidemiological studies carried out in China. Although most reports were fairly brief and complete information on covariates was not available, the results tended to support the potential for fluoride-mediated developmental neurotoxicity at relatively high levels of exposure in some studies. We did not find conclusive evidence of publication bias, although there was substantial heterogeneity among studies. Drinking water may contain other neurotoxicants, such as arsenic, but exclusion of studies including arsenic and iodine as co-exposures in a sensitivity analysis resulted in a lower estimate, although the difference was not significant. The exposed groups had access to drinking water with fluoride concentrations up to 11.5 mg/L (Wang SX et al. 2007); thus, in many cases concentrations were above the levels recommended (0.7–1.2 mg/L; DHHS) or allowed in public drinking water (4.0 mg/L; U.S. EPA) in the United States (U.S. EPA 2011). A recent cross-sectional study based on individual-level measure of exposures suggested that low levels of water fluoride (range, 0.24–2.84 mg/L) had significant negative associations with children’s intelligence (Ding et al. 2011). This study was not included in our meta-analysis, which focused only on studies with exposed and reference groups, thereby precluding estimation of dose-related effects.

The results suggest that fluoride may be a developmental neurotoxicant that affects brain development at exposures much below those that can cause toxicity in adults (Grandjean 1982). For neurotoxicants such as lead and methylmercury, adverse effects are associated with blood concentrations as low as 10 nmol/L. Serum fluoride concentrations associated with high intakes from drinking water may exceed 1 mg/L, or 50 µmol/L—more than 1,000 times the levels of some other neurotoxicants that cause neurodevelopmental damage. Supporting the plausibility of our findings, rats exposed to 1 ppm (50 µmol/L) of water fluoride for 1 year showed morphological alterations in the brain and increased levels of aluminum in brain tissue compared with controls (Varner et al. 1998).

The estimated decrease in average IQ associated with fluoride exposure based on our analysis may seem small and may be within the measurement error of IQ testing. However, as research on other neurotoxicants has shown, a shift to the left of IQ distributions in a population will have substantial impacts, especially among those in the high and low ranges of the IQ distribution (Bellinger 2007).

Our review cannot be used to derive an exposure limit, because the actual exposures of the individual children are not known. Misclassification of children in both high- and low-exposure groups may have occurred if the children were drinking water from other sources (e.g., at school or in the field).

The published reports clearly represent independent studies and are not the result of duplicate publication of the same studies (we removed two duplicates). Several studies (Hong et al. 2001; Lin et al. 1991; Wang SH et al. 2001; Wang SX et al. 2007; Xiang et al. 2003; Zhao et al. 1996) report other exposures, such as iodine and arsenic, a neurotoxicant, but our sensitivity analyses showed similar associations between high fluoride exposure and the outcomes even after these studies were excluded. Large tracts of China have superficial fluoride-rich minerals with little, if any, likelihood of contamination by other neurotoxicants that would be associated with fluoride concentrations in drinking water. From the geographic distribution of the studies, it seems unlikely that fluoride-attributed neurotoxicity could be attributable to other water contaminants.

Still, each of the articles reviewed had deficiencies, in some cases rather serious ones, that limit the conclusions that can be drawn. However, most deficiencies relate to the reporting of where key information was missing. The fact that some aspects of the study were not reported limits the extent to which the available reports allow a firm conclusion. Some methodological limitations were also noted. Most studies were cross-sectional, but this study design would seem appropriate in a stable population where water supplies and fluoride concentrations have remained unchanged for many years. The current water fluoride level likely also reflects past developmental exposures. In regard to the outcomes, the inverse association persisted between studies using different intelligence tests, although most studies did not report age adjustment of the cognitive test scores.

Fluoride has received much attention in China, where widespread dental fluorosis indicates the prevalence of high exposures. In 2008, the Ministry of Health reported that fluorosis was found in 28 provinces with 92 million residents (China News 2008). Although microbiologically safe, water supplies from small springs or mountain sources created pockets of increased exposures near or within areas of low exposures, thus representing exposure settings close to the ideal, because only the fluoride exposure would differ between nearby neighborhoods. Chinese researchers took advantage of this fact and published their findings, though mainly in Chinese journals and according to the standards of science at the time. This research dates back to the 1980s, but has not been widely cited at least in part because of limited access to Chinese journals.

In its review of fluoride, the NRC (2006) noted that the safety and the risks of fluoride at concentrations of 2–4 mg/L were incompletely documented. Our comprehensive review substantially extends the scope of research available for evaluation and analysis. Although the studies were generally of insufficient quality, the consistency of their findings adds support to existing evidence of fluoride-associated cognitive deficits, and suggests that potential developmental neurotoxicity of fluoride should be a high research priority. Although reports from the World Health Organization and national agencies have generally focused on beneficial effects of fluoride (Centers for Disease Control and Prevention 1999; Petersen and Lennon 2004), the NRC report examined the potential adverse effects of fluoride at 2–4 mg/L in drinking water and not the benefits or potential risks that may occur when fluoride is added to public water supplies at lower concentrations (0.7–1.2 mg/L) (NRC 2006).

In conclusion, our results support the possibility of adverse effects of fluoride exposures on children’s neurodevelopment. Future research should formally evaluate dose–response relations based on individual-level measures of exposure over time, including more precise prenatal exposure assessment and more extensive standardized measures of neurobehavioral performance, in addition to improving assessment and control of potential confounders.

## Supplemental Material

(94 KB) PDF
