# Supplementary material for: Developmental Fluoride Neurotoxicity: A Systematic Review and Meta-Analysis
Source: Environ Health Perspect. 2012 Jul 20;120(10):1362–8. doi: 10.1289/ehp.1104912 (PMC3491930; doi:10.1289/ehp.1104912)
Supplement: (94 KB) PDF [file ehp.1104912.s001.pdf]

Supplemental Material

Developmental Fluoride Neurotoxicity: A Systematic Review and Meta-Analysis

Anna L Choi, Guifan Sun, Ying Zhang, Philippe Grandjean

Supplemental Material, Table S1. Information on studies that were excluded

Reference, Study location	No. in high exposure group	No. in reference group	Age range (years)	Fluoride exposure: Assessment	Fluoride exposure: Range	Outcome Measure	Results	Reason for Exclusion
Liu et al. 2000 Tianjin, China	60	58	10-12	Drinking water	3.15 mg/L (high) 0.37 mg/L (reference)	CRT-RC[a]	Children in the high fluoride area scored significantly lower IQ scores than those in the control area	Duplicate study of Lu et al. 2000
Xu and Hu 1993 Ningxia, China	395	608	8-14	Drinking water	1.8 mg/L (high) 0.8 mg/L (reference)	Chinese Binet	No effect of high fluoride levels on IQ	Duplicate study of Xu and Hu in 1991
Calderon et al. 2000 Mexico	61 (total)	-	6-8	Drinking water; Urine	1.2-3 mg/L; 4.3 mgF/g creatinine	WISC-RM, Rey Osterreith-Complex Figure; CPT	Urinary F correlated positively with reaction time and inversely with visuospatial scores; IQ scores not influenced by fluoride exposure.	Individual-level measure of exposure
Rocha-Amador et al. 2007 Mexico	132 (total)	-	6-10	Drinking wáter; Urine	0.8-9.4 mg/L (means); 0.6-25 mg F/g creatinine	WISC-RM	An inverse association between F (in urine and in drinking water) and performance, verbal, and full IQ scores	Individual-level measure of exposure
Ding et al. 2011 Inner Mongolia, China	331 (total)	-	7-14	Drinking water; Urine	0.24-2.84 mg/L; 0.1-3.55 mg/L	CRT-RC[a]	Urine fluoride was inversely associated with IQ in the multiple regression model	Individual-level of exposure
Hu and Yu 1989 Shaanxi, China	198	181	6-14	Drinking water	7ppm (high); <0.8ppm (reference)	IQ (test not specified)	Effects of fluoride and IQ in children not specifically mentioned (but no effect of fluoride poisoning on intellectual ability in adults was reported)	Missing SDs in each group
Qin et al. 1990 Hebei, China	141 (high)	147 (normal) 159 (reference)	9-10.5	Drinking water	2.1-4 mg/L (high) 0.5-1 mg/L (normal) 0.1-0.2 mg/L (reference)	Raven	Fluoride levels can disrupt intellectual development	Missing mean (SD) of outcome measures
Xu and Hu 1991 Ningxia, China	395	608	7-17	Drinking water	3.99 mg/L (high); 0.73 mg/L (reference)	Chinese Binet	No effect of high fluoride levels on IQ	Missing SD of outcome measures
Li et al. 1993 Guizhou, China	-	-	8-13	Coal burning	2.69(1.32) mg/L (high); 2.01(1.11) mg/L (normal); 1.81(0.33) mg/L (low); 1.02(0.13) mg/L (reference)	CRT-RC	IQ scores were significantly lower among children in high and normal fluoride exposure areas than those in low and control areas	Missing number of subjects in each group
Hao et al. 2002 Henan, China	1346	1566	8-12	Drinking water	2.7-4.8 mg/L (high) <0.8 mg/L (reference)	CRT-RC[a]	Children from high F areas scored lower IQ than those from the control area	Missing mean(SD) of outcome measures
Wang et al. 2005 Guizhou, China	176	50	7-12	Urine	>1.0-8.6 mg/L (high) 0.58-1.0 mg/L (reference)	Raven	Children from the high fluoride group showed retarded development	Missing mean(SD) of outcome parameters
Trivedi et al. 2007 India	89	101	12-13	Drinking water;	5.55(0.42) mg/L (high); 2.01 (0.009) mg/L (reference)	IQ Questionnaire[b]	Children in the high fluoride area scored lower IQ than those from reference fluoride area	SDs of mean outcome parameter were questionably small

[a]CRT-RC denotes Chinese Standardized Raven Test, rural version (Wang et al. 1989)
[b]Developmed by Professor JH Shah (Desai K, Desai H. Psychological Measurement, Gujarat University Press, Gujarat State; India 1989)

References

Calderon J, Machado B, Navarro M, Carrizales L, Ortiz MD, Diaz-Barriga F. 2000. Influence of fluoride exposure on reaction time and visuospatial organization in children. Epidemiol 11(4):S153.

Ding Y, Gao Y, Sun H, Han H, Wang W, Ji X, Liu X, Sun D. 2011. The relationships between low levels of urine fluoride on children's intelligence, dental fluorosis in endemic fluorosis areas in Hulunbuir, Inner Mongolia, China. 2011. J Hazard Mater 186:1942-1946.

Hao K, Chen H, Zhu X. 2002. Research on the effect of high fluoride exposure on children's intelligence. Henan Med Info. 10(17):87. (in Chinese)

Hu Y, Yu Z. 1989. Research on the intellectual ability of 6-14 year old students in an area with endemic fluoride poisoning. Collection of papers and abstracts of 4th China Fluoride Research Association. 6:73. Available online: http://www.fluoridealert.org/chinese/.

Li SS, Ji JL, Kao YC. 1993. Comparison and analysis of the intelligence of children in different fluorosis communities. Chinese J Control Endemic Dis 8(6):372-373. (in Chinese)

Liu S, Lu Y, Sun Z, Wu L, Lu W, Wang X, Song Y. 2000. Report on the intellectual ability of children living in high fluoride zones. The Chinese Journal of Control of Endemic Disease. 200. 15(4):231-232. (in Chinese) (Also available: Fluoride 33(2):74-78, which was included in the analysis).

Qin L, Cui S, Chen R, Chang Y. 1990. Using the Raven's Standard Progressive Matrices to determine the effects of the level of fluoride in drinking water on the intellectual ability of school-age children. Chinese J Control Endemic Dis 5(4):203-204. Available online: http://www.fluoridealert.org/chinese/.

Rocha-Amador D, Navarro ME, Carrizales L, Morales R, Calderon J. 2007. Decreased intelligence in children and exposure to fluoride and arsenic in drinking water. Cad. Saúde Pública 23 Suppl 4:S579-587.

Trivedi MH, Verma RJ, Chinoy NJ, Patel RS, Sathawara NG. 2007. Effect of high fluoride water on intelligence of school children in India. Fluoride 40(3):178-183.

Wang S, Zhang H, Fan W, Fang S, Kang P, Chen X, Yu M. 2005. The effects of endemic fluoride poisoning caused by coal burning on the physical development and intelligence of children. J Appl Clin Pediatr 20(9):897-899. (in Chinese) (Also available: Fluoride 41(4):344-348).

Xu K, Hu X. 1991. The analysis of the effects of high fluoride exposure to children's physical and intellectual development. J Ningxia Med Sch. 13(4):19-22. (in Chinese)

Xu K, Hu X. 1993. The analysis of the effects of high fluoride exposure to children's physical and intellectual development. Endemic Dis Bull. 8(2):92-95.

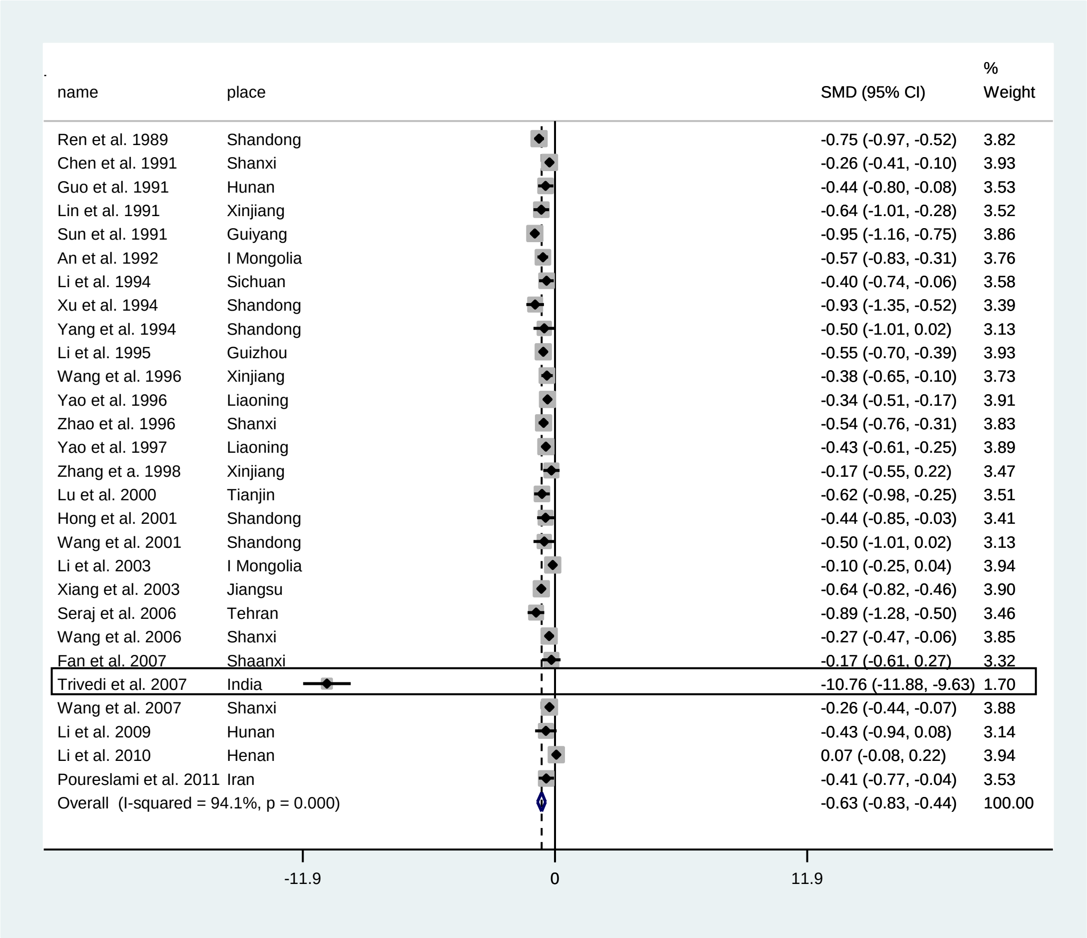

Supplemental Material, Figure S1. Random-effect SMD estimates and 95% CIs of child's intelligence score associated with high exposure to fluoride among 28 studies including Trivedi et al. 2007 with questionably small SDs (highlighted in a black textbox). SMDs for individual studies are shown as solid diamonds (♦), and the pooled SMD is shown as a non-filled diamond (◊). Horizontal lines represent 95% CIs for the study-specific SMDs.
